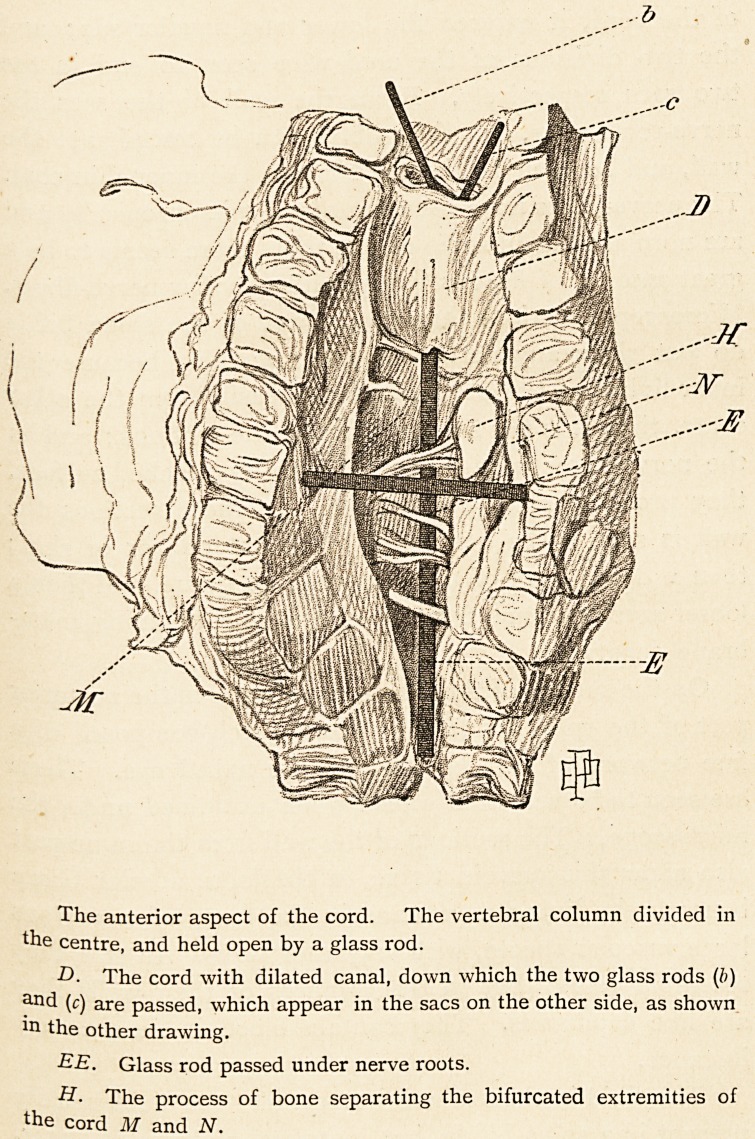# On a Rare Form of Spina Bifida (Syringo-Myelocele)

**Published:** 1892-03

**Authors:** Charles A. Morton

**Affiliations:** Registrar, Bristol General Hospital; Surgical Registrar and Pathologist to the Bristol Hospital for Sick Children and Women


### ON A RARE FORM OF SPINA BIFIDA (SYRINGO-MYELOCELE).

BY

CHARLES A. MORTON, F.R.C.S. Eng.,

*Registrar, Bristol General Hospital; Surgical Registrar and Pathologist to the Bristol Hospital for Sick Children and Women.*

F. G., aged four months, was admitted into the Children's Hospital in April, 1891, under the care of Mr. Norton, to whose kindness I am indebted for permission to record the case.

A markedly pedunculated spina bifida existed in the lumbo-sacral region. It was as large as an ostrich's egg, and only partially covered with skin. In places the membranous wall was very thin. The head was abnormally large, and the interparietal fissure open. No craniotabes was present. The child seemed blind, and squinted in with the right eye. The right leg was twisted outwards below the knee-joint, and there was marked talipes calcaneus and convexity of the sole. The other leg was normal. No movement was observed in either lower limb.

On the 18th, thirty minims of Morton's fluid were injected into the sac, the baby being kept in the horizontal position.

On the evening of the 19th the temperature went up to 102°. There was no leaking from the sac.

At five a.m. on the 20th the child was found to be comatose, but not convulsed. When examined at ten a.m. it was still unconscious, and had occasional slight laryngeal stridor. There was external squint in the eye that before squinted in. Extreme tension of the anterior fontanelle was present, so that it felt almost as hard as the surrounding bone. The pulse was 160. The child only lived a few hours after these observations were made.

Unfortunately, permission could not be obtained for a general *post mortem*, but only to examine the spine. The anterior fontanelle lost its extreme tension after death; and pressure on the spina bifida in no way altered the tension there.

On opening the spina bifida it was found to consist of two quite separate sacs, the upper one being the larger. They appeared to communicate with the central canal by very small openings, and resembled meningoceles. But on opening up the vertebral canal from the other side, through the bodies of the vertebræ, the cord was found to have an enormously dilated central canal, and to bifurcate—the two parts being separated by a small bony projection—and on passing a probe down the dilated central canal and along one of the divisions beyond the

The spina bifida laid open from behind, showing the large sac *A* and the small sac *B*.

The glass rod (*a*) holds the walls of the sac open. Passing into the sac underneath the rod (*a*) and at right angles to it is another rod [marked (*c*) in the other drawing] which goes down the dilated central canal. (*b*) is the rod also marked (*b*) in the other drawing passed from the central canal into the sac *B*. The glass rod (*c*) in this drawing has been represented passing through the lower margin of the inferior sac as if it were a nerve root.

The anterior aspect of the cord. The vertebral column divided in the centre, and held open by a glass rod.

*D*. The cord with dilated canal, down which the two glass rods (*b*) and (*c*) are passed, which appear in the sacs on the other side, as shown in the other drawing.

*EE*. Glass rod passed under nerve roots.

*H*. The process of bone separating the bifurcated extremities of the cord *M* and *N*.

point of bifurcation, it entered the upper larger sac; and when passed through the canal in the other portion of the cord, it entered the lower and smaller sac; and the two divisions of the cord were seen to end in the two sacs respectively. From the wall of the sacs the nerve-roots arise, and their fibres can be traced into the wall, and be seen to lie just beneath the inner thin coat. Ths nerve-fibres have been examined microscopically and are normal; but in some of the roots there seems to be a great amount of fibrous tissue, with very few nerve-fibres. Microscopic sections through the wall of the spina bifida do not show any nerve-elements, probably because the inner lining of the sac separates so easily from the other coats, that it is not seen in most of the sections; and as the nerve-fibres were connected with its attached surface, they probably separated with it. In these sections the wall is seen to be composed of an outer layer of skin; then a dense layer of thick connective tissue, and then a looser areolar tissue from which the smooth lining membrane separated.

On the surface of this lining membrane in the posterior part of the spina bifida is a brown patch composed of a fine network of vessels visible to the naked eye. These are seen to be very thin-walled when examined under the microscope. The sections of the wall from the same part also show this nævoid tissue, in the loose areolar tissue beneath the lining membrane in the form of numerous large vascular spaces, with very thin walls. In size they resemble veins; but in the thinness of their walls they are like capillaries. They contain blood in nearly every instance.

The fluid in the sac was watery, like the fluid usually present in a spina bifida, but contained no trace of sugar.

This form of spina bifida (syringo-myelocele) in which the sac is formed by a dilatation of the central canal of the cord is very rare. Out of 125 cases examined by the Committee of the Clinical Society, there were only two cases. I have examined the specimens of spina bifida in the College of Surgeons' Museum, and cannot find a single specimen of it there.

The complete division of the sac into two is also rare. Incomplete division is, however, not uncommon; there are several such specimens in the College Museum.

Attention should also be directed to the bifurcation of the cord, and the separation of the two halves by the bony process.

The position of the cord in the vertebral canal is what we often find in spina bifida—it occupies the position of fœtal life; *i.e.*, the whole length of the canal. In this case the spina bifida is lumbo-sacral, and yet it is formed by the cord, which passed down the whole length of the vertebral canal.

The evidence that this specimen is a syringo-myelocele consists in the fact that the central canal ends in the sac, and that the nerve-roots arise in and course through the wall of the sac, and do not cross its cavity as in a myelocele.[1]

Its causation is of some interest. Not only must there have been a failure of the mesoblastic plates to close over the cord, as happens in all cases of spina bifida, but the neural canal can never have become obliterated by the growth of the nerve-elements of the cord. Consequently, we should not expect much nervous tissue in the wall of the sac, or the nerve-roots arising from it, a condition

[1] The specimen is in the Museum of the Children's Hospital, where I shall be glad to show it to anyone interested in it.

already referred to in connection with this case. The connective tissue found so abundantly in the roots would, on the other hand, probably own a mesoblastic origin, and might be present when the nerve-fibres of epiblastic origin might be deficient. Again, as the medullary sheath is not formed until after the axis cylinders, there may be nerve-fibres formed only of axis cylinders, both in the sac-wall and nerve-roots, which have not gone on to the development of medullary sheaths, and so are not demonstrable by osmic acid in the sections.

There is one other point which, although it does not elucidate the pathology, yet is of interest from its bearing on treatment. The opening of the large sac of the spina bifida into the central canal was obstructed by a little fine lymph, from the injection of Morton's fluid—the early stage of a possible cure, had the child lived. Clinically, there was nothing to show the presence of the two sacs, and yet, of course, only one could be injected. This may explain the diminution in size or cure of a large portion, but not the whole of a spina bifida after injection.

## Figures and Tables

**Figure f1:**
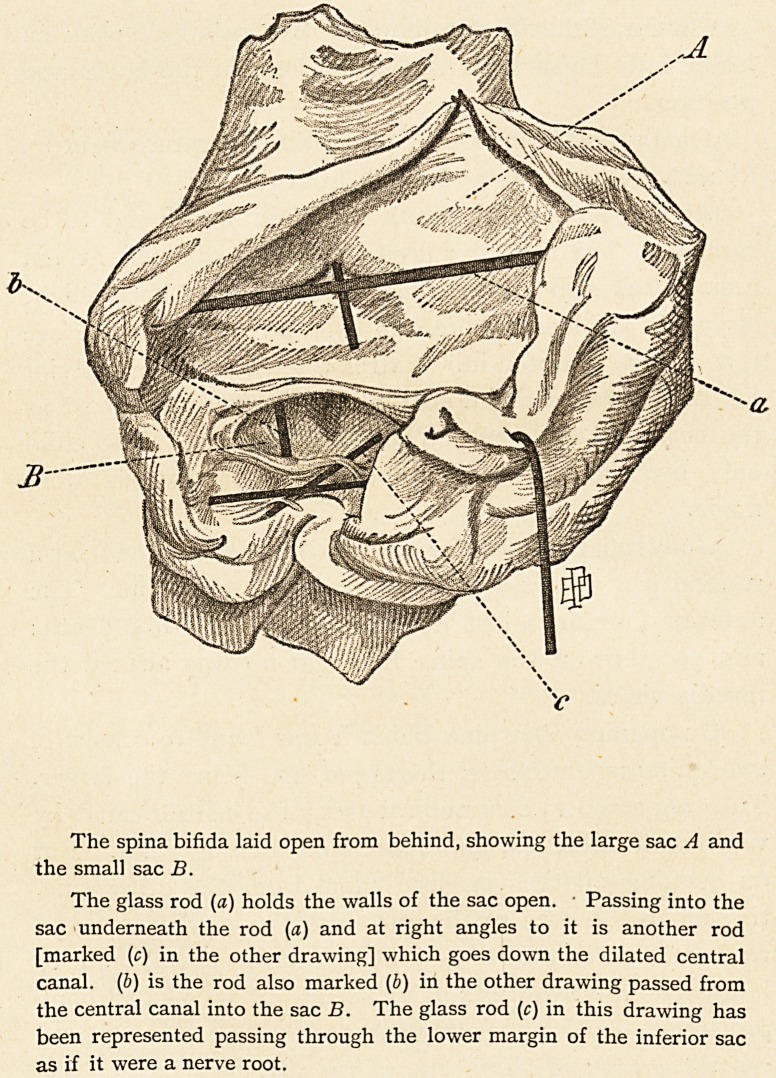


**Figure f2:**